# Cytoreduction surgery and hyperthermic intraperitoneal chemotherapy for treating advanced peritoneal metastases of hepatocellular carcinoma

**DOI:** 10.1515/pp-2019-0030

**Published:** 2020-05-15

**Authors:** Kuo-Chen Hung, Kun-Lin Yang, Guan-Cheng Huang, Yu-Fu Chen, Wen-Teng Chang, Chia-Chang Chuang

**Affiliations:** Department of Trauma Surgery, Kaohsiung Chang Gung Memorial Hospital, Chang Gung University and College of Medicine, Kaohsiung 83301, Taiwan; Department of Animal Science, National Peimen Agriculture and Industrial school, Tainan, Taiwan; Division of Hemato-oncology, Department of Internal Medicine, Yuan’s General Hospital, Kaohsiung, Taiwan; Department of Education and Research, Yuan’s General Hospital, Kaohsiung, Taiwan; Department of Biological Science and Technology, Chung Hwa University of Medical Technology, Tainan, Taiwan; Department of Emergency Medicine, National Cheng Kung University Hospital, College of Medicine, National Cheng Kung University, Tainan, Taiwan; Hyperthermic Center, Department of Surgery, Yuan’s General Hospital, Kaohsiung 802793, Taiwan

**Keywords:** cytoreduction surgery, hepatocellular carcinoma, hyperthermic intraperitoneal chemotherapy, peritoneal metastasis

## Abstract

**Background:**

An effective treatment strategy for peritoneal metastasis (PM) of hepatocellular carcinoma (HCC-PM) has yet to be established. Although cytoreductive surgery (CRS) and hyperthermic intraperitoneal chemotherapy (HIPEC) have shown favorable outcomes in certain malignancies, their role in peritoneal metastatic HCC is unclear. Herein, we present a series of patients with HCC-PM treated with CRS/HIPEC and evaluate their outcomes.

**Methods:**

Records of patients with HCC-PM who had undergone CRS/HIPEC at the Hyperthermia Center of Yuan’s General Hospital, Kaohsiung, Taiwan, between September 2015 and December 2016 were reviewed retrospectively. Patients were followed up until September 2019. We assessed the clinical courses and outcomes of these patients to clarify the benefits of CRS/HIPEC.

**Results:**

Six patients were included in our study. HCC-PM occurred synchronously in one patient and occurred metachronously in five patients after therapeutic minimally invasive procedures, including radiofrequency ablation, laparoscopic hepatectomy, robotic hepatectomy or spontaneously. The median peritoneal cancer index was 18.5. All patients experienced complete peritoneal cytoreduction without perioperative mortality. One patient had two CTCAE grade 3 complications. The median follow-up was 16 months. The median overall survival was 15.7 months. Four patients died of lung metastasis or liver failure owing to intrahepatic recurrence. The survival rates observed at 1, 2, and 4 years were 66.7%, 33.3%, and 33.3%, respectively.

**Conclusions:**

CRS followed by HIPEC is feasible in patients with HCC-PM and might provide selected patients a chance for local disease control and longer survival. CRS/HIPEC might be considered as a treatment option in highly selected patients, as part of multimodal therapy approaches.

## Introduction

Hepatocellular carcinoma (HCC) is the fifth most common malignancy worldwide and the second most common cause of cancer-related deaths [1]. In Taiwan, HCC is the fourth leading cancer. According to 2014 statistics published in Taiwan, the age-adjusted incidence of HCC is 32.7 per 100,000 persons (Taiwan Cancer Registry. Cancer registry annual report. 2014. http://www.hpa.gov.tw/Pages/Detail.aspx?nodeid=269&pid=7330). The occurrence of peritoneal metastases (PMs) in HCC at resection is rare (0.8%) [2].

Traditionally, PM has been considered a sign of advanced HCC. The prognosis of patients with peritoneal metastatic HCC (HCC-PM) is generally dismal with median survival between 2.1 and 12.5 months [3]. However, several studies have reported that aggressive surgical resection of peritoneal tumors may prolong survival in certain patients [4, 5, 6].

Although cytoreductive surgery (CRS) combined with hyperthermic intraperitoneal chemotherapy (HIPEC) has shown favorable outcomes in certain cancers with PMs [7, 8, 9], its role in HCC-PM is unclear. Herein, we report a series of patients with advanced HCC-PM treated using CRS and HIPEC.

## Patients and methods

Records of patients with HCC-PM who had undergone CRS/HIPEC at the Hyperthermia Center of Yuan’s General Hospital, Kaohsiung, Taiwan, between September 2015 and December 2016 were retrospectively reviewed. The patients were followed up until September 2019. This study received IRB approval and informed consent was obtained from all patients.The inclusion criteria for patients receiving CRS/HIPEC were as follows: having resectable peritoneal tumors, manageable or resectable intra-hepatic HCC, Child’s A liver disease, good Eastern Cooperative Oncology Group (ECOG) performance (0–1), and adequate renal and heart function.After the initial evaluation, a further contrast-enhanced cross-sectional imaging study, namely a computed tomography (CT) scan or magnetic resonance imaging (MRI), of the chest, abdomen, and pelvis was performed to quantify peritoneal disease burden and exclude extra-abdominal spread.All patients had undertaken abdominal surgery before, so we did not use staging laparoscopy strategy. A midline laparotomy was performed, and the entire abdomen was explored. The peritoneal extent of the disease was measured using the peritoneal cancer index (PCI), which ranges from 0 to 39 [10]. The completeness of cytoreduction was recorded using the Jacquet and Sugarbaker classification system [10].After CRS, HIPEC was performed using the closed-abdomen technique.Mitomycin C was the chemotherapeutic agent used in our patients because of its heat-stability and well-established pharmacokinetics in HIPEC [11]. Another reason to use MMC is that it is effective in transarterial chemoembolization for HCC [12]. After surgery, the patients were admitted to the intensive care unit for at least one postoperative day until both cardiac and pulmonary functions stabilized.Surgical complications were graded according to the National Cancer Institute Common Terminology Criteria for Adverse Events v4.0 [13]. Perioperative mortality was defined as any death within 30 postoperative days or during the same hospitalization.The surveillance process involved a physical examination, complete blood count test, blood chemistry test, serum alpha-fetoprotein measurement, and contrast-enhanced CT scan (or MRI) of the chest, abdomen, and pelvis every 3 months. Recurrence was diagnosed based on clinical, radiological, or histological findings and was consistently confirmed in multidisciplinary team meetings. For patients receiving more than one CRS/HIPEC procedure, survival analyses were performed from the date of the first procedure. Overall survival (OS) was defined as the time from the first CRS/HIPEC procedure to the date of death or the date of the last follow-up. Median survival times and survival rates were computed using the Kaplan-Meier method.

## Results

Between September 2015 and December 2016, eight patients with HCC-PM were referred to Yuan’s General hospital for CRS/HIPEC. Two of the eight patients did not meet the inclusion criteria and were excluded from this study. The remaining six patients underwent CRS/HIPEC. Synchronous HCC-PM occurred in one patient and metachronous HCC-PM in 5 patients after spontaneous rupture of HCC and minimally invasive procedures including radiofrequency ablation (RFA), laparoscopic hepatectomy, and robotic hepatectomy. The clinical data are summarized in Table 1.

**Table 1:** Clinical characteristics and outcomes of patients with HCC-PM undergoing CRS/HIPEC.

Variables	#1	#2	#3	#4	#5	#6	Median [range]
Age, years	64	56	66	35	66	69	66 [35–69]
Gender	M	M	F	F	F	M	
Virus	HBV	HBV	HCV	HBV	HBV	HBV	
ECOG status	0	0	0	0	0	0	
ICG at 15 min	5.8	7.8	x	6.1	29.8	2.7	
Liver cirrhosis/Child-Pugh Score	N	N	N	N	Y/A	N	
Previous intervention before CRS/HIPEC	CRS	RobH	LapH	DxLp	LapH	RFA	
Sorafenib treatment	Failed	Failed	Failed	Failed	Failed	No treatment	
Type of PM	M	M	M	S	M	M	
Interval (last intervention to CRS/HIPEC, mo)	14	5	8	3	11	10	9 [3–14]
Interval (PM diagnosis to CRS/HIPEC, mo)	3	3	3	3	6	1	3 [1–6]
**Operative data**							
Operative time, min	810	865	585	1065	380	735	772.5 [380–1065]
EBAL, mL	2204	1755	250	880	150	570	725 [150–2204]
Transfusion	Y	N	N	Y	N	N	
PCI	20	22	17	25	8	16	18.5
CC score (0,1,2)	0	0	0	0	0	0	
Organs resected	Om, Gb, Li	Om, Pr, Li, Sp, Gb, Aw	Aw, Li, Pr, Om	Sp, Gb, Om, Ov, App, Pr, Li, PV-TT	Om, Pr	Pr, Om, Gb, Li, App	
Intraoperative complication	N	N	N	N	N	N	
**Outcome data**							
Hospital stay, days	14	25	19	20	24	18	19.5
Morbidity (CTCAE Grade 3–4)	0	0	2	0	0	0	
Mortality (90 days)	N	N	N	N	N	N	
Recurrence after CRS/HIPEC	Li, Pr, abLN	Li, Bone	Li, Lung, Bone	Li, Lung, abLN	Li, PV-TT	Li, Pr	
Treatment for hepatic recurrences	TACE	HAIC, Hep	HAIC	HAIC	TACE	Hep	

ICG, Indocyanine Green test; Y, yes; N, no; RobH, robotic hepatectomy; DxLp, diagnostic laparoscope; LapH, laparoscopic hepatectomy; RFA, radiofrequency ablation; PM, peritoneal metastasis; S, synchronous; M, metachronous; EBAL, estimated blood and ascites loss; PCI, peritoneal cancer index; CC, completeness of cytoreduction; Om, omentum; Gb, gallbladder; Li, liver; Pr, peritoneum; Sp, spleen; Aw, abdominal wall trocar site; Ov, ovarian; PV-TT, portal vein tumor thrombus; App, appendix; abLN, abdominal lymph node; TACE, transarterial chemoembolization; HAIC, hepatic arterial infusion chemotherapy.

The median time interval from PM diagnosis to CRS/HIPEC was 3 (1–6) months. The median time interval from the previous procedure to CRS/HIPEC was 9 (1–14) months. The median age at the time of CRS/HIPEC was 65 (35–69) years. Although five patients had received sorafenib, the treatment failed. All patients had hepatic viral diseases (HBV or HCV), a well-preserved liver function (Child A liver disease or better), and an adequate performance status (ECOG 0). The operative data and outcome are presented in Table 1.

The median intraoperative PCI was 18.5 (8–25). Complete peritoneal macroscopic cytoreduction (CC0-1) was achieved in all patients. Concurrent intrahepatic metastases, which did not show in the preoperative CT study, were identified intraoperatively in two patients. Multivisceral resection (≥3 organs) including hepatectomy was required in 5 patients. The omentum was the most common site of the spread.

Two grade 3 adverse events were observed in one patient, which were a symptomatic right pleural effusion and midline wound dehiscence on the 30th day postoperatively. No perioperative mortality was observed. At the last follow-up (September 2019), two patients were alive. The alive two patients had oligo peritoneal recurrence and then had received further peritoneal tumor resection. All patients had hepatic recurrences (Table 1). The post-recurrence treatment included hepatic arterial infusion chemotherapy (HAIC) for multiple hepatic nodules, and hepatectomy or transarterial chemoembolization (TACE) for the solitary tumor according to the HCC treatment guideline of Yuan’s hospital. Four patients had died without peritoneal recurrence according to the image examination (CT/MRI) conducted 2–3 months before their death. One patient had died 4 months after CRS/HIPEC due to progressive intrahepatic metastases with portal vein tumor thrombosis (PV-TT) and liver failure. Two patients had died of respiratory failure owing to lung metastases. One patient had died of upper gastrointestinal bleeding owing to progressive intrahepatic metastasis and liver failure.

**Figure 1:**
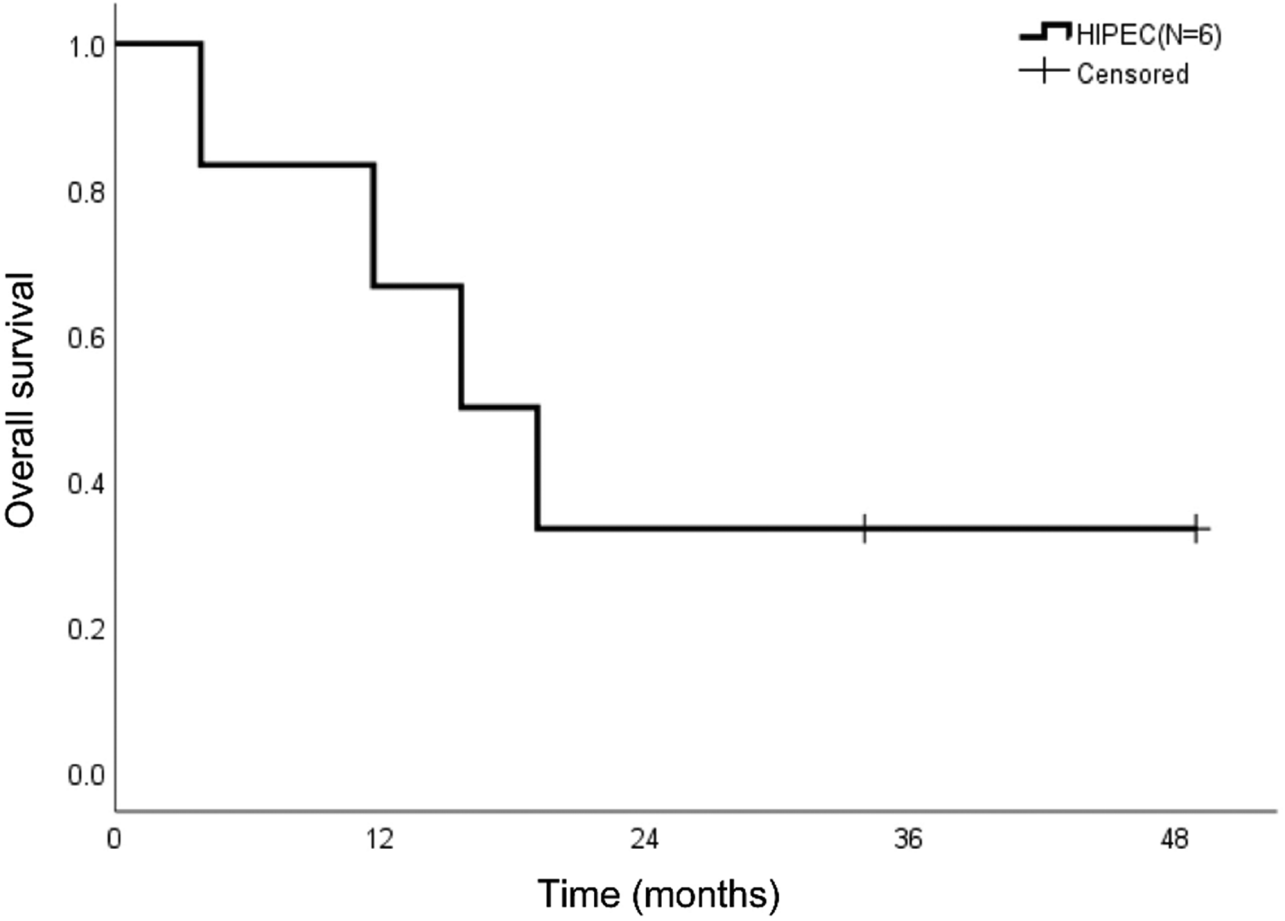
Survival rate of patients with peritoneal HCC after CRS/HIPEC. The survival rate of patients with peritoneal HCC after CRS/HIPEC was 66.7%, 33.3%, and 33.3% at 1 year, 2 years, and 4 years, respectively.

The median follow-up was 16 months. The median OS was 15.7 months. The survival rates observed at 1, 2, and 4 years were 66.7%, 33.3%, and 33.3%, respectively (Figure 1). All patients had recurrences. The recurrent treatment modality included transarterial chemoembolization, radiation therapy, hepatic artery infusion chemotherapy, hepatectomy or tumor resection which depended on the recurrent sites.

**Figure 2:**
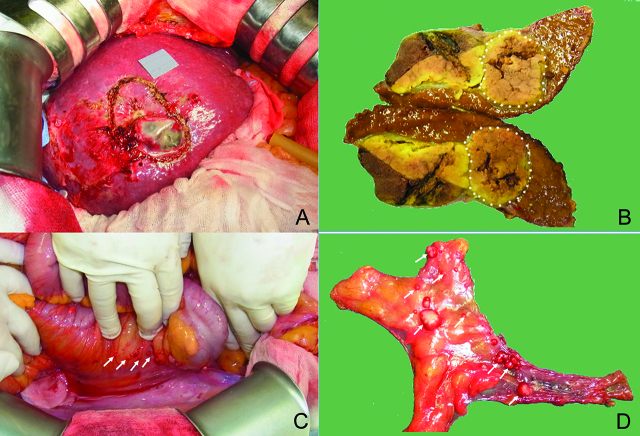
Patient #6 tumor location and later peritoneal recurrence. Tumor location for RFA in patient #6 was subcapsular at S5-8 (A, B); HCC peritoneal nodules distributed diffusely on the right paracolic gutter (arrows) (C, D).

**Figure 3:**
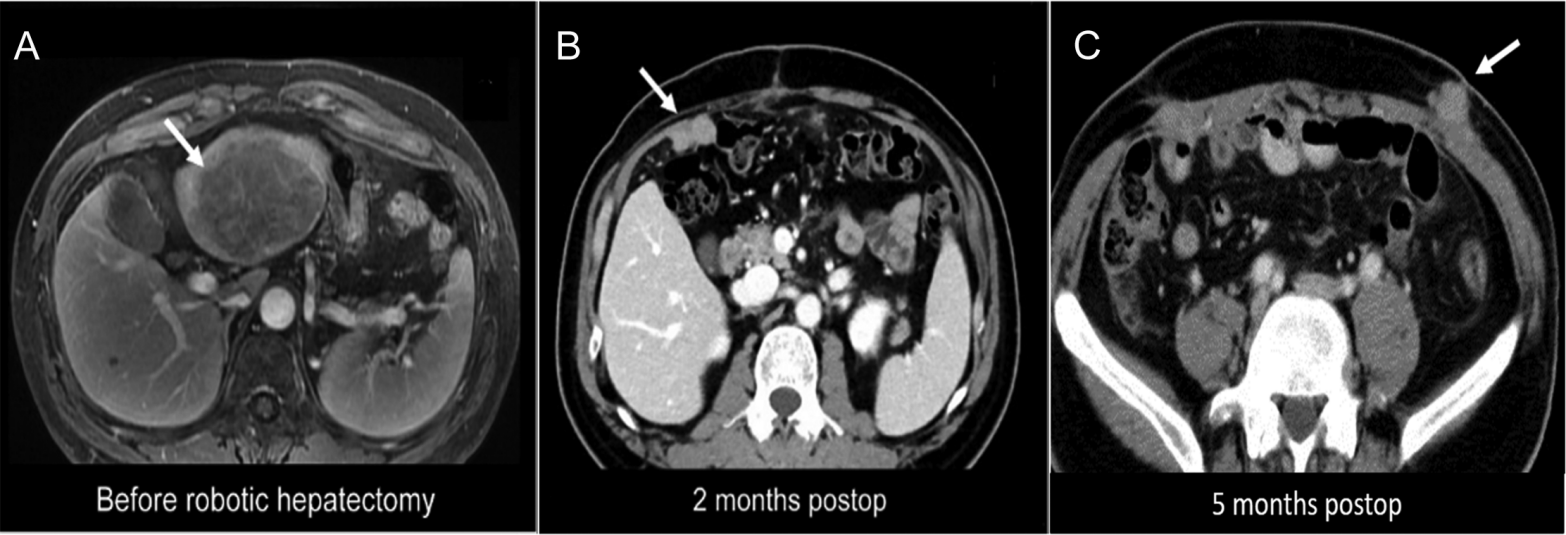
Patient #2 tumor location and late port site metastases. Imaging results for patient #2, who had undergone robotic hepatectomy for a 7.3-cm HCC in S2-3 (A); CT imaging within 2 months postoperatively showed a new 2.8-cm tumor in the right subhepatic region (B); CT imaging within 5 months postoperatively revealed port site metastasis at the left abdominal wall (C).

**Figure 4:**
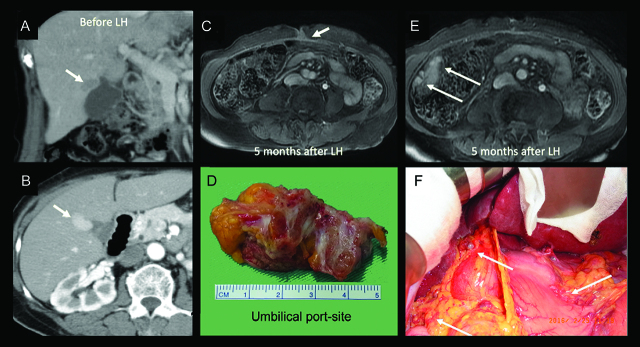
Patient #3 tumor location and late port site, subhepatic metastases. Results for patient #3, who had undergone laparoscopic hepatectomy (LH) for a 2.5 cm HCC in S5 (A, B); MRI imaging conducted within 5 months postoperatively and operative findings showed umbilical port site metastasis (C, D) and multiple nodules (1 cm in size) in the right subhepatic region (arrows) (E, F).

The tumor location for RFA in patient #6 was subcapsular at S5-8, and diffusely HCC nodules on the peritoneum occurred 9 months after RFA (Figure 2). Three patients with one solitary HCC (T1) occurred advanced PMs within 3–6 months after laparoscopic or robotic hepatectomy; two of these three patients had port site metastases (Figures 3 and 4).

## Discussion

Generally, HCC-PM is rare and considered an incurable terminal disease. Herein, we report a series of six patients with HCC with advanced PMs who were treated using CRS/HIPEC. The major findings in these patients were the lack of postoperative morbidity and the effective control of peritoneal disease. Therefore, the patients, who would otherwise have been considered for symptomatic treatment only, became ideal candidates for additional therapeutic interventions and consequently had a chance for improved survival.

Patients with HCC-PM are classified as having advanced stage disease (C) according to the Barcelona-Clinic Liver Cancer grading system; for such patients, a palliative treatment is indicated [14]. In the palliative setting, the survival of patients treated with sorafenib and/or systemic chemotherapy has been reported to be between 6 and 13.6 month [15, 16, 17, 18].

Although no consensus exists regarding the therapeutic management in HCC-PM, aggressive CRS may be of survival benefit in patients with localized HCC-PM, with an observed survival of more than 24 months [5]. Careful selection of patients with localized peritoneal disease for CRS, with the consideration of the performance status, liver function, and tumor biology, may lead to a successful outcome in patients with HCC-PM [19].

Since the 1980s, a novel treatment has emerged: combining CRS, for macroscopic disease treatment, with HIPEC, for microscopic residual disease treatment [20]. This therapeutic strategy has changed the treatment of several visceral malignancies with PM; it is currently regarded as the standard of care for pseudomyxoma peritonei from appendiceal cancer, and peritoneal mesotheliomas [9, 21].

CRS/HIPEC used to treat colorectal PM has been successful [22, 23]. However, the randomized trial PRODIGE 7 presented in June 2018 at ASCO meeting failed to show the benefit of HIPEC with 30 min oxaliplatin in colorectal PM treatment [24]. The negative results might change clinical practice in the future. More research is needed to determine which patients are still benefiting from receiving HIPEC or other forms of intraperitoneal drug delivery in addition to CRS [25].

In the past two decades, several studies have also reported that CRS/HIPEC improved survival of patients with ovarian cancer PM [26, 27, 28, 29] and patients with gastric cancer [30, 31, 32].

Although the role of CRS/HIPEC in HCC-PM remains unknown, several studies have reported the application of CRS/HIPEC for HCC-PM treatment [33, 34, 35, 36, 37]. Two studies have demonstrated CRS/HIPEC to be associated with a superior survival trend compared with CRS only [35, 36]. Tabrizian et al. [35] reported 14 patients with HCC and limited PM, of whom seven received CRS and seven received CRS/HIPEC. The mean PCI was 9.9 ± 8.3, and complete CRS (CCR 0–1) was achieved in all but one patient. The median OS of the entire cohort (CC 0–1) and of patients who received CRS/HIPEC (CC 0–1) were 35.6 and 42.1 months, respectively. Moreover, the 3-year recurrence rate after CRS was 100%.

Berger et al. reported 22 patients with HCC-PM treated with CRS; of the 22 patients, 5 received additional treatment involving HIPEC. The median OS for all patients was 23.6 months. Patients treated with HIPEC had a better median OS than did those treated with CRS alone (29.7 vs 19.5 months); however, this survival difference was not statistically significant [36]. Recently, Mehta et al. reported an international multicentric cohort with 21 patients undergoing CRS/HIPEC for HCC-PM of HCC. The median PCI was 14. The median OS was 46.7 months. The projected 3-year and 5-year OS were 88.9 and 49.4% respectively. PCI less than 15 was a favorable factor for better recurrence free survival [34].

PCI, the extent of peritoneal disease, is a major prognostic factor in several malignancies with PM, such as colorectal cancer, epithelial ovarian cancer, and gastric cancer [31, 38, 39]. In general, a high PCI value is an independent adverse prognostic factor. In this study, the median PCI was 18.5, which is higher than those in other studies [34, 35, 37].

Intraperitoneal cancer cell from the primary tumor that could be spontaneous or iatrogenic [40, 41]. In this study, one patient was synchronous PM. Four patients were metachronous PM after therapeutic minimally invasive interventions.

RFA has a risk of needle tract implantation and PM. Stigliano et al. [42] reported the median risk of metastasis, defined as metastasis occurring in either the subcutaneous tissue or peritoneal cavity, was 0.61% (0%–5.56%) for RFA without biopsy and 0.95% (0%–12.5%) for RFA with biopsy. Llovet et al. [41] indicated that subcapsular tumor location was one of risk factors for neoplastic seeding, like the lesion of patient #6 in this study (Figure 2).

Laparoscopic or robotic hepatectomy has been applied to treat HCC. Recent studies have revealed that these minimally invasive procedures are as safe as the open procedure, and the long-term results are also inspiring [43, 44, 45]. In this study, advanced PM or port site metastases occurred following laparoscopic or robotic hepatectomy for the solitary HCC. Although the incidence is very uncommon, this issue has rarely been mentioned in the literature. Three papers have reported a total of only 4 cases involving port site or PM following laparoscopic hepatectomy [45, 46, 47].

The main limitations of this study are the small number of cases and the retrospective nature of the study. There may be a strong selection bias since this cohort represents a highly selected group of patients.

Cancer treatment is the multidisciplinary strategy. CRS/HIPEC is considered palliative. Patients with PM often die of intestinal obstruction due to tumor invasion of the intestine or malnutrition due to malignant ascites. Therefore, palliative treatment of PM can reduce intestinal obstruction or malignant ascites, thereby improving the quality of life and nutritional status of patients, giving patients the opportunity to receive other treatments, and possibly increasing survival.

## Conclusions

CRS followed by HIPEC is feasible in patients with HCC-PM and might provide selected patients a chance for local disease control and longer survival. CRS/HIPEC might be considered as a treatment option in highly selected patients, as part of multimodal therapy approaches.
